# Individual Osmotic Fragility Distribution: A New Parameter for Determination of the Osmotic Properties of Human Red Blood Cells

**DOI:** 10.1155/2014/162102

**Published:** 2014-01-02

**Authors:** Tomasz Walski, Ludmiła Chludzińska, Małgorzata Komorowska, Wojciech Witkiewicz

**Affiliations:** ^1^Institute of Biomedical Engineering and Instrumentation, Wrocław University of Technology, Wybrzeże Wyspiańskiego 27, 50-370 Wrocław, Poland; ^2^Regional Specialist Hospital in Wrocław, Research and Development Centre, Kamieńskiego 73a, 51-124 Wrocław, Poland

## Abstract

The aim of our experiments was to characterise and to validate the osmotic fragility test when applied to human blood samples with no significant alterations of osmotic fragility but with a differentiating shape of the haemolysis curve. All experiments were carried out on human erythrocytes taken from the Regional Centre of Blood Donation and Blood Therapy in Wrocław. The washed erythrocytes were exposed to near-infrared radiation (NIR) or ozonated, and the osmotic fragility test was applied. The osmotic fragility, calculated from the experimental haemolysis curve for the control and cells irradiated for 15 min, is the same within the empirical error. Calculation of the first derivative of the haemolysis curve allowed us to visualise the changes in osmotic fragility distribution after exposure to NIR. By contrast, significant changes both to the osmotic fragility value and the distribution of osmotic properties were observed after an erythrocytes ozonation procedure. Description of cell osmotic properties requires at least two parameters—the value of osmotic fragility and the slope of the haemolysis curve in the region where absorbance sharply increases due to cell haemolysis.

## 1. Introduction

The osmolality of mammalian blood plasma is maintained in the 270–310 mosmol range. The major substances regulating the osmotic properties are cations, for example, sodium (136–145 mM) and potassium (3.6–5.4 mM), and anions, for example, chloride and hydrogencarbonate. The molar concentrations of other osmotically active metabolites, such as urea, glucose, and plasma proteins, are low, but they account for 8–12 mosmol colloid oncotic pressure [1]. The degree of resistance of red blood cells (RBC) to lysis as a result of a decrease in the NaCl concentration of their environment is the basis of the osmotic fragility test. Experimentally, the conventional osmotic fragility test consists of measuring the intensity of light transmitted through a Hb solution produced by suspension of erythrocytes in a hypotonic media. The light wavelength commonly used is *λ* = 540 nm, where only haemoglobin, as a major protein of the RBC, contributes to light absorption. Osmotic fragility is defined by shifts in the haemolysis curve, which relates absorbance versus NaCl concentration, and is often established by determination of 50% of the haemolysis points.

Osmotic fragility is widely used to elucidate mechanisms of the influence of different factors on the osmotic properties of RBC membranes, such as shear stress and mechanical haemolysis [2], temperature [3], ultrasound effects [4], drugs [2], and irradiation [4]. The osmotic fragility test is also useful for diagnosis of certain haematological diseases, for example, haemolytic anaemia, hereditary spherocytosis, and elliptocytosis, glucose-6-phosphate dehydrogenase deficiency, and sickle cell anaemia, as well as for RBCs from uremic or diabetic patients [5, 6]. Low osmotic resistance may lead to intravascular haemolysis, which causes a reduction of the RBC life span [5, 7]. The osmotic fragility curve of red cells not only reflects the average membrane and cytoplasmic properties, but may also provide information on the distribution of those properties within the sample [8]. 

### 1.1. Theoretical Consideration

Haemoglobin is released through haemolytic pores formed in the stretched membranes of swollen spherical cells in a hypotonic medium. Cell lysis occurs immediately after swelling to its spherical shape. During osmotic haemolysis, a RBC becomes a sphere with a maximum volume of 1.5 to 1.6 times higher than the discocyte volume in an isotonic medium [9]. Swelling is a limiting process of cell lysis; thus the osmotic fragility reflects the ability of the cell to uptake water in a hypotonic medium. When the external osmotic pressure is reduced, the cell volume increases. The relation between osmotic pressure and cell volume is expressed by the equation [6]:

(1) $$\begin{array}{c} \frac{\prod_{}}{\prod_{\text{iso}}}=\frac{V_{\text{iso}}-b}{V-b}, \end{array}$$

where *b* is a part of the RBCs volume osmotically inactive and ∏_iso_ and *V*_iso_ are the osmotic pressure and volume for the isotonic media and ∏ and *V* for hypotonic media. The critical osmotic pressure ∏_*cj*_ for an individual cell, *j*, and the critical volume *V*_*cj*_ corresponding to it are rewritten in the following form [6]:

(2) $$\begin{array}{c} \overset{*}{\underset{c,j}{\prod }}=\frac{\prod_{cj}}{\prod_{\text{iso}}}=\frac{V_{\text{iso},\text{j}}-b_{j}}{V_{c,j}-b_{j}}. \end{array}$$

A distribution of the critical volumes associated with the distribution of critical osmotic pressures is expected. The absolute values of osmotically nonresponsible water are about 3.57 and 0.33 g water/g dry mass in the haemoglobin solution and in the erythrocytes, respectively [10]. It was proposed that the extent of osmotically nonresponsive water correlates with water interacting with protein. Protein-water interaction is a key factor in the restriction of water diffusion within the cell. Bogner et al. demonstrated that about 20% of intracellular water is directly related to intracellular proteins in terms of osmosis in human erythrocytes [10].

The shape of the haemolysis curve, which shows the degree of cell lysis as a function of salt concentration, results from the erythrocytes' membranes strength distribution function [11, 12]. The authors assumed that the strength distribution function *f*_*d*_ is Gaussian in form and is expressed by the following equation:

(3) $$\begin{array}{c} f_{d}=\frac{1}{2}\exp\left[{\left(\frac{c-p_{1}}{p_{2}}\right)}^{2}\right], \end{array}$$

where *c* is NaCl concentration and *p*_1_ and *p*_2_ are parameters characterising the location of the maximum of the distribution function and its dispersion, respectively. Parameters *p*_1_ and *p*_2_ are positive and nonzero [11]. The fraction of erythrocytes having strengths greater than value *x* is given by the ratio of integrals as follows:

(4) $$\begin{array}{c} \frac{\int_{x}^{\infty }f_{d}dc}{\int_{-x}^{\infty }f_{d}dc}=\frac{1+\mathrm{erf}\left(\left(p_{1}-x\right)/p_{2}\right)}{2}, \end{array}$$

where *erf*(*x*)  is the error function with argument  *x*.

Finally, based on (4), the fraction of haemolysed RBCs, *H*_*f*_, at a given stress *x* (critical NaCl concentration) can be further calculated in terms of the complimentary error function:

(5) $$\begin{array}{c} H_{f}=\frac{1}{2}\mathrm{erf}\;c\left(\frac{x-p_{1}}{p_{2}}\right). \end{array}$$

When haemolysis is measured using an absorbance at 540 nm, a new parameter *p*_3_ is introduced:

(6) $$\begin{array}{c} H_{\text{abs}}=p_{3}\mathrm{erf}\;c\left(\frac{x-p_{1}}{p_{2}}\right), \end{array}$$

where *p*_3_ is one-half of the maximum absorbance, *p*_2_ is the ratio of NaCl concentration in which haemolysis occurs, and *p*_1_ is the osmotic fragility (an NaCl concentration where 50% of the cells are haemolysed). The above mentioned parameters could be visible on the first derivative of the haemolysis curve. Troiano et al. identified the presence of two populations of *Iguana iguana* red cells through analysis of the osmotic fragility derivative curve [8].

### 1.2. Aim of the Study

In this work, we discuss the shape of the haemolysis curve and its relationship to the distribution of individual RBC osmotic fragility. We studied the effect of NIR radiation on the osmotic properties of erythrocytes. Preliminary experiments have shown that the values of osmotic fragility are within the same empirical error for nonirradiated and irradiated cells; however, the shape of the haemolysis curve changed markedly.

The purpose of our study was to characterise and to validate the osmotic fragility test when applied to blood samples with no significant alterations of osmotic fragility but with a differentiating shape of the haemolysis curve.

We suggest that a complete description of cell osmotic properties requires at least two parameters: the osmotic fragility value and the slope of the haemolysis curve in the region where absorbance increases due to cell haemolysis. As an example of the usefulness of this approach, we provide a report on the results for RBCs exposed to NIR radiation and exposed to an ozone atmosphere in order to extend the knowledge of the effects of photobiostimulation and oxidative damage on erythrocytes. The proposed procedure is complementary to the information gained from the haemolysis curve.

## 2. Materials and Methods

### 2.1. Preparative Procedure

Experiments were carried out on human erythrocytes not older than 10 days from donation and which were taken as RBC concentrates (RBCC) from the Regional Centre of Blood Donation and Blood Therapy in Wrocław (RCBDT). Units preserved in citrate phosphate dextrose adenine (CPDA-1) with the addition of Adsol (contains adenine, glucose, mannitol, and sodium chloride) were kept refrigerated at 277 K until the day of measurement. The erythrocytes were then isolated by centrifugation at 1750 ×g for 240 seconds at 277 K, washed three times in a phosphate-buffered saline (PBS) of pH = 7.4, and diluted in PBS to obtain a 10% haematocrit.

### 2.2. Irradiation Procedure

To investigate the effect of NIR, the suspension of cells in PBS was exposed to the radiation of a halogen lamp equipped with a 700–2000 nm filter. Samples were kept in a dark box, and light was focused on a flat glass tube (15 cm^3^ volume, 10% haematocrit) containing the suspension. The power density of incident light was 6.9 mW·cm^−2^. During exposure, the suspension was gently stirred and cooled. Irradiation temperature was kept at a constant 293 ± 2 K by means of an air- and water-cooling system. The cells were irradiated for 900 sec periods.

### 2.3. Ozonation Procedure

Ozone was generated from gaseous oxygen using a Sorbios Ozone Generator model GSG 001.2 (Sorbios GMbH, Germany) with a flow rate of 5 or 10 dcm^3^/h. The stirred suspension (15 cm^3^ volume with 10% haematocrit) of the red cells in the PBS solution was incubated for 900 sec under a stream of the ozone/oxygen mixture (0.5% v/w). The cells were then washed and resolved in the PBS solution again.

### 2.4. Osmotic Fragility Curve

The osmotic fragility (OF) test measures the amount of released haemoglobin in samples of blood placed in equal quantities in a series of tubes containing an aqueous solution of sodium chloride. The series of tubes contain different concentrations of the sodium chloride solution from isotonic to a low ionic strength close to that of distilled water (0–145 mM NaCl buffered by a 10 mM phosphate buffer, pH = 7.4). The ionic strength exerted within each tube generates different levels of red cell haemolysis. Thirty minutes after the incubation of human RBCs in the sodium chloride solution, the suspension was centrifuged at 1750 ×g for 240 seconds. The obtained supernatant was examined spectrophotometrically (Nicolet Evolution 60, Thermo Scientific). The amount of released haemoglobin, which is proportional to the lysed cell number, was estimated by colorimetric analysis at 540 nm, which is one of the haemoglobin spectrum maxima. The haemolysis curve was measured twice: firstly for rough estimation of the osmotic fragility and secondly with high precision determination, where the sample absorbance increases sharply with the decreasing of NaCl concentration (the steepest part of the curve). All absorbance values were normalised using (7), that is, 0%—no haemolysis occurred and 100%—all cells are haemolysed:

(7) $$\begin{array}{c} A_{N}=\frac{A_{x}-A_{\text{iso}}}{A_{aq}-A_{\text{iso}}}100\%, \end{array}$$

where *A*_*N*_ is normalised relative absorbance, *A*_*x*_ is the absorbance of the solution for the measured sample, *A*_iso_ is the absorbance of the solution in an isotonic external medium, and *A*_*aq*_ is the absorbance when 100% of the cells haemolysed in distilled water. The region of the haemolysis curve beginning at the point where haemolysis of the cells begins until the end of the process (the steepest part of the curve) can be estimated as linear. The concentration of the external solution when 50% of the cells are haemolysed is a measure of the osmotic fragility parameter (OF).

**Figure 1:**
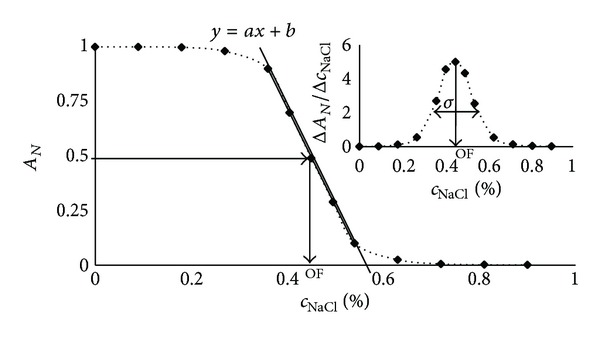
Example of the haemolysis curve and the parameters discussed in the text: *σ*—dispersion of the osmotic properties within the human RBC sample, *a*—the slope of the steepest part of the haemolysis curve, and OF—the osmotic fragility.

The distribution of osmotic properties was calculated as the first derivative of the haemolysis curve. The analysis of the first derivative of the haemolysis curve can be used for distinguishing the distribution of osmotic properties within the RBC population [9]. In relation to the first derivative curve (distribution of osmotic properties in the measured cell population), we estimated the dispersion (*σ*) of the osmotic properties within the RBC sample. The example of an ideal haemolysis curve and the parameters discussed above are presented in Figure 1.

### 2.5. Effect of Storage Time

The effect of the storage time of RBCs on NIR radiation effectiveness in the alteration of the erythrocyte's osmotic properties was also investigated. In this case, haemolysis curves were carried out on RBCC aged 5 to 42 days. The erythrocytes were isolated and irradiated as described in Sections 2.1 and 2.2.

### 2.6. Statistical Analysis

The obtained results were analysed using the statistical package *Statistica 8*, produced by *StatSoft*. Student's paired *t*-test was used for the statistical analysis. Differences were considered significant at *P* < 0.05.

## 3. Results and Discussion

**Figure 2:**
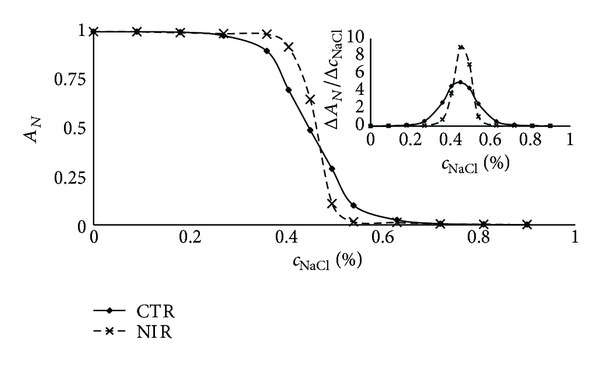
Haemolysis curves for control and NIR irradiated human erythrocytes. The upper right figure shows the distribution of osmotic properties calculated from the first derivative of the haemolysis curve. The mean osmotic fragility for modified and unmodified cells is nearly the same; the position of the maximum of the first derivative curve is not shifted.

Figure 2 shows the haemolysis curve obtained for the control sample and the irradiated sample. The osmotic fragility, calculated from that curves, is the same within the empirical error. Calculation of the first derivative of the function presented in Figure 2 allowed us to visualise the changes in osmotic fragility distribution after exposure to NIR. Because the osmotic fragility for modified and unmodified cells is nearly the same, the maximum position on the first derivative curve is not shifted, but the distribution of cells that differentiate due to their osmotic properties is more dispersed for the control cells. After irradiation, the cells are more unified by their osmotic properties. It is clear that cells with the lowest and the highest osmotic fragility after NIR modification are closer to the mean value of this parameter. The overall analysis of haemolysis curves and first derivative curves of the studied blood samples is shown in Table 1. For all the samples studied, no significant differences between the mean osmotic fragilities for the control and irradiated samples were observed (*P* > 0.05), but the distribution of the osmotic properties of treated cells is always less dispersed (*P* < 0.0001).

**Figure 3:**
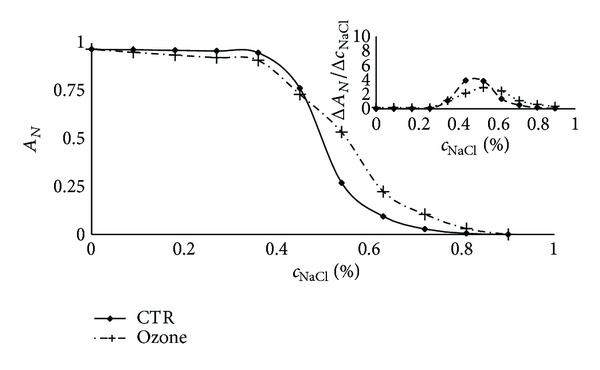
Haemolysis for control and ozone treated human erythrocytes. The upper right figure shows the distribution of osmotic properties calculated from the first derivative of the haemolysis curve. The mean osmotic fragility increases, and the osmotic properties are much more widely dispersed in comparison to the control samples. A nonzero value of the distribution function for the isotonic solution is a consequence of spontaneous haemolysis of cells in the isotonic solution.

The oxidative stress induces a reverse effect. The effect of ozone on RBC osmotic properties is shown in Figure 3. Osmotic fragility increases; that is, the value of the osmotic fragility is shifted to a higher NaCl concentration, after oxidative stress, and the shape of the haemolysis curve and the osmotic fragility distribution function are changed. In contradiction to the irradiated cells, the osmotic properties of ozonated cells are much more widely dispersed in comparison to the control samples. Exposure of RBCs to ozone resulted in an increased osmotic fragility due to lipid and protein peroxidation [13], which also induces spontaneous haemolysis of cells (autohaemolysis) in an isotonic solution. A nonzero value of the distribution function for the isotonic solution is a consequence of this process.

**Figure 4:**
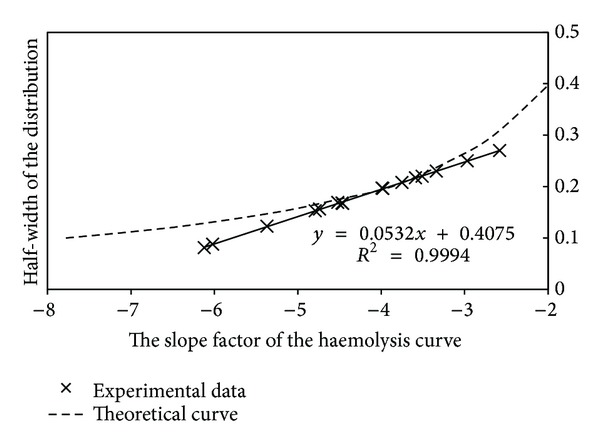
Dependence between the width of the osmotic fragility distribution and the slope factor of the haemolysis curve for experimental data along with computer simulations for ideal Gaussian distributions. The experimental data fits the theoretical curve only in some part. The high and low values of mean osmotic fragility show that the properties of the cells' osmotic distribution do not have a Gaussian character.

Figure 4 illustrates the dependence between the width of osmotic fragility distribution and the slope of the steepest part of the haemolysis curve. The experimental dependence between these parameters is linear (Figure 4) for all blood samples measured by us. Theoretical simulation for ideal Gaussian distribution clearly shows that the relation between the width of distribution and the slope is only linear within a narrow domain. For high and low values of mean osmotic fragility, the obtained results show that the properties of the cells' osmotic distribution do not have Gaussian distribution. Thus, a full description of the osmotic properties of the cell population requires at least two parameters—the osmotic fragility value and the width (dispersion *σ*) of osmotic fragility distribution. While the dispersion *σ* is a linear function of the steepness of the haemolysis curve, a new parameter has been introduced—the slope factor of the haemolysis curve, which is a measure of distribution of the osmotic properties in the cell population.

**Figure 5:**
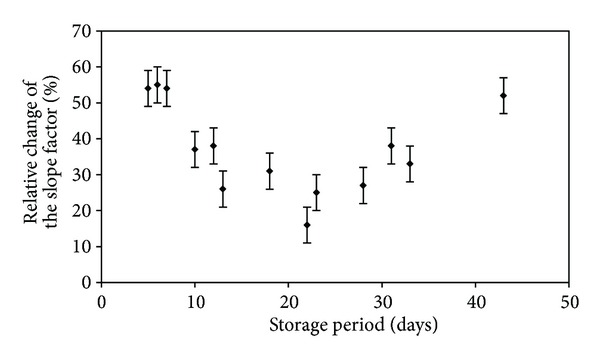
Relative change (in percentage) of the slope factor of the haemolysis curve for NIR irradiated cells compared to the control as a function of the erythrocyte storage period. Erythrocytes stored during shorter and longer periods and the dispersion of distribution decreased around 50%–60%, while the minimal value was reached between 10 and 30 days of storage.

There is a question concerning the mechanism of the decrease of osmotic fragility distribution after exposition to NIR. Several pieces of evidence show that erythrocytes [14–16], liposomes [17, 18], and even molecules such as amino acids [19, 20] or DNA [20] are sensitive to irradiation with NIR light. In our previous papers, we reported the effects of NIR irradiation (*in vitro*) on erythrocyte membranes. Changes of membrane structure and polarity, shape, and the viscoelastic properties of erythrocytes were monitored. After exposure to NIR, the membrane fluidity decreases, the polarity decreases in the vicinity of the polar heads, the rate of haemolysis is decreased from the control value, and the zeta potential, electrophoretically measured, was changed upon irradiation as well as the cell shape. Erythrocytes after exposure to near infrared radiation (NIR), and which were later ozonated, are protected against oxidative stress. All the processes observed [14–20] could be induced by the changes of water structures, which weaken the interactions between the water molecules and membrane surfaces and strengthens the hydrophobic effects. In relation to protein 4.1, a component of the red cell membrane skeleton exists in two major forms: 4.1a and 4.1b. It was established that the mean ratio of both proteins (4.1a/4.1b) in the erythrocytes correlates with the average life span [21]. The measurements of the cell age parameter revealed that erythrocytes of different ages were not uniformly affected by cell swelling. The progressive decrease in K^+^_i_ and increase in Na^+^_i_ contents in a subpopulation of the RBCs of increasing cell density are consistent with the decrease in the cell volume and with cell water loss. On the other hand, the decreased surface area (as a result of the formation of hemichromes) and increased cytoplasm viscosity (due to increased mean cell haemoglobin concentration) lower cell deformability [22, 23], which is observed in cell aging. In the densest cells, the mean sphericity (a dimensionless measure of surface-to-volume) is not significantly different than for other cell populations, but the distribution variance of the oldest population is significantly increased. This may be evidence of the loss of volume regulation and increased membrane permeability of these RBCs [22]. This is supported by the results of Marks and Johnson, who found that young mature human erythrocytes were more resistant to haemolysis in hypotonic media than old cells [24]. Thus, the youngest and the oldest cells represent the tails of the distribution function. The narrowing of the osmotic fragility distribution is probably caused by rehydration of the youngest and dehydration of the oldest RBCs. It is clear that both of the groups of cells are the most sensitive to NIR radiation. If we assume that *in vitro *aging is comparable to *in vivo* aging, the results presented in Figure 5 strongly support our suggestions. We investigated the effect of the storage time of RBCs on NIR radiation effectiveness in the alteration of the erythrocytes' osmotic properties. Erythrocytes stored for a shorter (<10 days) and longer (>30 days) time after exposure to NIR were characterised by decreased dispersion of the distribution (approx. 50%–60%) as compared to the control samples. Between 10 and 30 days of storage, the relative change of the slope factor reached a minimal value of 10%–20%.

## 4. Conclusion

For a more accurate description of the haemolysis curve, a new parameter has been introduced—the slope factor of the haemolysis curve, which is a linear function of the distribution of the osmotic properties in the measured cell population.

Using the proposed interpretation of the curves of haemolysis, we demonstrated that NIR most efficiently works on both young and old cells.

## Figures and Tables

**Table 1:** Effect of near infrared radiation (NIR) on red blood cell osmotic properties, described by distribution parameters.

Haemolysis curve parameter	Control	NIR	*P* value
	mean ± SD	mean ± SD	
Osmotic fragility (%)	0.39 ± 0.03	0.40 ± 0.03	0.78
Slope factor of haemolysis curve (%^−1^)	−3.65 ± 0.68	−4.88 ± 0.92	0.000015
Half-width of the distribution (%)	0.214 ± 0.036	0.148 ± 0.049	0.000010

*n* = 8, values of *P* < 0.05 were considered significant.
